# Vikings, LISA and pain killers…

**DOI:** 10.1038/s41390-025-04528-6

**Published:** 2025-11-03

**Authors:** Egbert Herting

**Affiliations:** Department of Pediatrics, University of Lübeck, Lübeck, Germany

Until some decades ago, there was doubt whether premature newborns could feel pain at all. Asking parents and nurses (e.g. using pain scores as in the current paper,^1^ but also evaluation of the infant´s physiological reaction to noxious insults), has clearly answered this question by now. Yes, they can!

Premature babies undergo hundreds of potentially painful procedures during their stay on a neonatal intensive care unit (NICU). Minimal handling strategies avoiding e.g. prolonged mechanical ventilation, or non-invasive monitoring devices avoiding unnecessary venipunctures, are just examples how clinical practice has changed in the last 20 years in the best interest of our small patients.

Of course, pharmacological pain management seems to be a most convenient option. However, simply the enormous number of drugs that have been named in this context demonstrates clearly that the ideal substance for this purpose in newborns has never been found. On the contrary, especially substances used for sedation as midazolam or phenobarbital, are seen with some concern today as both animal experimental data and clinical follow-up seem to indicate potential harm on a rapidly developing brain. On the other hand, the potential life-long effects of pain/stress on the NICU are currently being discussed as well. A true dilemma!

Morphine and derivatives like fentanyl (used in the current study) are amongst the favorites analgetics on a NICU. Effects and side effects are known for years in adults; data on premature infants are however still scarce. A recent review article^2^ pointed out that the ideal analgetic drug should reach receptors e.g. in the brain very rapidly, should have a short duration of action, allowing exact titration of the dose, and should not interfere with spontaneous breathing by suppression of the respiratory drive and/or chest rigidity. Not to speak of longer-term negative effects on e.g. gut mortality, synaptogenesis or brain development. Simply, the ideal analgetic drug does not exist in neonatology.

Especially for morphine, the interval between the injection and the start of laryngoscopy in clinical life is often too short. In this way the analgetic effects for the premature baby may be minimal and the drug given in that way at most “soothes the bad conscience” of a neonatologist performing a potentially painful procedure on a neonate.” In addition, the fear of side effects often leads to use of (too) low doses that are clearly inefficient to achieve adequate analgesia. In this context, comfort care and close observation of the infant as done in the current study, are of prime importance anyhow to define the need of analgesia and to titrate the right dose. In this way, the study focuses on the individual needs of the infants which has a long tradition in the Scandinavian countries.

Avoidance of mechanical ventilation and promoting CPAP use (CPAP = continuous positive airway pressure) was reality in Scandinavia already in the early nineties at a time when more than 80% of all babies below 1000 g routinely were ventilated via an endotracheal tube in the rest of the world. Admittedly, if you try CPAP a lot you will also experience CPAP failure. Already 30 years ago neonatal care was so centralized in Scandinavia that even a capital like Stockholm (30.000 deliveries) only had (and needed!) only one Level III that ventilated premature babies. Berlin, for example, with a similar number of deliveries, had 9 Level III units at that time! In this way CPAP failure in a Level II unit in Sweden or Denmark meant that the infants had or be transferred to a Level III centre. Avoiding stress and separation of mother and child was therefore also a strong driver for the development of new ideas. Respiratory distress syndrome (RDS) is the most common cause of CPAP failure in the most premature infants. Surfactant derived from animal sources was a causative treatment, but until that time was only given via an endotracheal tube during mechanical ventilation. Hendrik Verder was the first to publish surfactant administration via a small diameter tube during spontaneous breathing and CPAP in a Danish journal.^3^ Interestingly, the black and white picture in that publication showed a mother with a premature infant during skin to skin care, underlining the family centered approach of neonatology in the North of Europe. At the same time short intubation followed by Surfactant instillation and extubation (INSURE = INtubate Surfactant Extubate) was developed as alternative method and the thin catheter method was forgotten until it was rediscovered by Angela Kribs and colleagues in Cologne some 10 years later. By that time CPAP had also become popular in Germany at least in some centers so that the first 2 randomized studies were performed in the German Neonatal Network (GNN), demonstrating this approach helps to avoid mechanical ventilation. A variety of techniques and methods and names have been described^4^ until finally the name LISA^5^ (Less Invasive Surfactant Administration) “made the race”.

The insertion of a laryngoscope (even if advanced very gently and not too deep) and the insertion of a tube (however small it may be….) are for sure “unpleasant” (said at least) experiences. In this way discussion came up right away “how mean this can be” to perform LISA without (pre-) “medication”. Of note, the infants that received LISA in Germany were much more immature than the INSURE population in Scandinavia. It was noted that these tiny infants reacted much more sensitive to analgesia (suppression of spontaneous breathing) and also with hypotension especially when they received sedation as well. The procedure was often done soon after birth in the delivery suite and as birth per se is painful the “believers” in LISA without premedication propagated that there might be something like “perinatal analgesia” e.g. by high oxytocin levels present during delivery, creating a “window of opportunity” for performing LISA without drugs, but at the same time without too much stress for the infant. Personal observations were in line with what the first studies showed. Yes, analgesia reduces pain (scores) but at the same time interferes with spontaneous breathing^6,7^ and in this way endangers the overall success of the LISA method.

As observed in the current trial, there is already quite some discomfort in infants with RDS before the LISA procedure. The 2 largest LISA centers in Europe, Cologne and Vienna, soon propagated non-pharmacological comfort care (noise/light reduction, positioning, holding, swaddling, tucking, sucrose) and one initial attempt of LISA without analgesia. A concept also used in the current trial is Caffeine given to promote spontaneous breathing. Running trials including the current study evaluate if this “bundle” is really the right approach.

“Can non-pharmacological comfort care replace Fentanyl in LISA” is therefore a most relevant question. The first results of 17 infants randomized either to receive saline or fentanyl are described here.^1,8^ Using of the COMFORTneo score the effects were observed of infants that received non-pharmacological comfort care and saline as opposed to those receiving fentanyl. The authors conclude that pausing the procedure and improving non-pharmacological care was sufficient in most patients with scores ≥ 14, irrespective whether fentanyl or saline was given before. This is of course of a (too) small number of observations for any valid conclusion. Therefore, the main conclusion of the manuscript is certainly that the study design seems feasible. Feasibility and safety/data monitoring interim analyses are often part of the design of clinical studies. However, most often such results are handled confidentially only by a data/safety monitoring committee but not communicated to the investigators or even the public while the study is running to avoid bias. Yes, the study is blinded, but the “blinded” nurses were able to tell what drug or placebo the infants had received in about 70% of cases. This reminds of the attempts to blind surfactant studies 30 years ago e.g. with a separate dosing team, where also of course the experienced nurses knew exactly what was going on.

The authors conclude that the frequent use of naloxone (55% vs. 56%) in both groups may be an indicator that the blinding procedure effectively prevented performance bias. The simple truth however, could also be that Naloxone only has very limited effects. The data for premature infants are really sparse and similar to the use of atropine, the idea to use such drugs is more based on theoretical concepts than on real world data. Interestingly, the use both of atropine and of naloxone has steeply declined in premature infants in the Germany (GNN) in the last decade.

Other methods as surfactant nebulization avoiding the “unpleasant” manipulation in the mouth with a laryngoscope (video or standard) are not within easy reach at the moment.^9^ The “D” in RDS stands for distress and surfactant in itself is one way to help the infants in this situation! Timely and early administration is important!

Even with measures as deferred consent or a call for more participating NICUs^10^ only 17 infants were included during a 9 months study period. This seems to be the real “hurdle” that has to be overcome by the study team to reach the final goal. The author of this comment, and for sure also Mona Lisa with her very special smile, would love NONA-LISA (NON-pharmacologic Approach Less Invasive Surfactant Administration) to recruit the numbers needed to answer a most relevant question.

More details on study data are available through the corresponding author.
